# AGING, TASK DIFFICULTY, AND EFFORT: A META-ANALYSIS

**DOI:** 10.1093/geroni/igz038.2592

**Published:** 2019-11-08

**Authors:** Ryan Best, Laure Freydefont, Alexandra M Freund

**Affiliations:** University of Zurich, Zurich, Switzerland

## Abstract

Tasks of increasing difficulty require increasing levels of cognitive engagement from participants. The costs associated with cognitive engagement rise with age in response to normative cognitive decline. Additionally, previous studies have shown an interaction between age and task difficulty, with age differences in effort expenditure increasing along with task demands. Motivational accounts of effort allocation predict the opposite relationship, where increased task difficulty in the face of declining cognitive abilities result in disengagement among older adults, comparatively lowering their effort expenditure relative to younger adults that remain committed to the task. The current study quantitatively reviews the available literature on age and effort expenditure across tasks of increasing difficulty. An initial meta-analysis found no age differences in effort across task difficulty, but inspection of the significantly heterogeneous effect sizes indicated that measurement domain might account for some of the variance found between the effect sizes. A second, post-hoc meta-analysis was conducted, recoding effect sizes giving preference to subjective measures. Subsequent moderator variable analyses found that the combined effect of age and domain of effort measurement explained a sufficient portion of the variance across effect sizes. When using physiological measures, effort was not found to differ across task difficulty for either age group. Alternatively, when measured subjectively, effort was reported to greatly increase (>1 standard deviation) with difficulty, with a larger increase in younger adults. Results are discussed in terms of effort mobilization across adulthood and the importance of measurement domain in the interpretation of results.

